# The complexities of treating brain and spinal cord tumors: a review of current approaches

**DOI:** 10.1097/MS9.0000000000001213

**Published:** 2023-09-01

**Authors:** Abdallah Mansour, Abdullah Trefi, Majd Mansour, Abdullah Shekho, Shadi Salloum

**Affiliations:** a Tishreen University Hospital; b Faculty of Medicine, Tishreen University, Latakia, Syria; c Harvard Medical School, Boston, MA

**Keywords:** brain and spinal cord tumours, metastatic tumours, novel therapies, surgical therapies

## Abstract

This article provides an overview of brain and spinal cord tumours, including their types, diagnosis, and treatment approaches. Brain and spinal cord tumours are complex and can be caused by various factors. They can be divided into two main categories, primary and metastatic tumours, which present their own unique challenges and complexities when it comes to treatment. Diagnosing brain and spinal cord tumours requires a careful evaluation of the patient’s medical history and symptoms, as well as a variety of diagnostic tools and techniques. Treatment approaches include surgery, radiation therapy, and chemotherapy, each with its own benefits and drawbacks. The choice of treatment depends on the type and location of the tumour, as well as the patient’s individual needs and preferences. Despite advances in treatment, there is a pressing need for further research to improve the effectiveness and safety of these treatments.

## Introduction

Highlights

- Brain and spinal cord tumours are complex and can be caused by various factors, including genetic mutations, exposure to radiation, and environmental toxins.
- These tumours can be divided into two main categories: primary and metastatic tumours, each presenting unique challenges and complexities when it comes to treatment.
- Diagnosing brain and spinal cord tumours requires a careful evaluation of the patient’s medical history and symptoms, as well as a variety of diagnostic tools and techniques.
- Treatment approaches include surgery, radiation therapy, and chemotherapy, each with its own benefits and drawbacks. The choice of treatment depends on the type and location of the tumour, as well as the patient’s individual needs and preferences.
- Despite advances in treatment, there is a pressing need for further research to improve the effectiveness and safety of these treatments.

Brain and spinal cord tumours are a significant health concern, as they can have serious consequences for patients and their families^1^ These tumours can affect individuals of all ages and can be caused by a variety of factors including: genetic mutations, exposure to radiation, and environmental toxins^2^. Treating brain and spinal cord tumours requires a deep understanding of the underlying biology behind these tumours. Adding to the complexity of treatment, brain and spinal cord tumours are often located in vital and sensitive areas of the body that can be difficult to access. In recent years, significant advances in the treatment of brain and spinal cord tumours have been made, with notable breakthroughs in therapies, technologies, and approaches that have positively impacted patient outcomes. These advancements include refined surgical techniques, advanced radiation therapy methods, targeted therapies tailored to specific genetic mutations, and the emergence of immunotherapy as a promising treatment option^3^. These treatments can be used alone or in combination, depending on the individual patient and the specific characteristics of their tumour^4^. However, despite these advances, many patients still experience poor outcomes, and there is a pressing need for further research to improve the effectiveness and safety of these treatments^5^.

In this narrative review, we aimed to provide an overview of the epidemiology, diagnosis, and treatment options for brain tumours. To gather relevant information, we conducted a comprehensive search on the PubMed database using specific search terms. The inclusion criteria for selecting articles included a publication date between 2001 and 2023 and relevance to the topic of interest. We excluded articles that were not in English or did not meet the scope of our review. Additionally, we sought information from reputable organizations and references cited in the selected articles to complement the narrative. A total of 49 articles were reviewed.

Our objective is to comprehensively cover brain and spinal cord tumours, including their diagnosis and treatment options.

## Types of brain and spinal cord tumours

According to the American Cancer Society, brain and spinal cord tumours can be divided into two main categories: primary tumours, which originate in the brain or spinal cord, and metastatic tumours, which have spread to the brain or spinal cord from another part of the body^6^. Primary tumours can be further classified based on their location and the type of cells they originate from, among other factors. Some common types of primary brain tumours include gliomas (tumours that develop from glial cells), meningiomas (tumours that arise from the meninges, the protective membranes covering the brain and spinal cord), and pituitary tumours (tumours that form in the pituitary gland, a small gland at the base of the brain that regulates hormone production)^7^. These tumour types vary in prevalence and incidence, with gliomas being the most common primary brain tumours and meningiomas accounting for the majority of benign brain tumours^1^. Each type of brain or spinal cord tumour presents unique challenges when it comes to treatment. For example, the location of the tumour can affect the surgical approach that is used, and tumours that are close to critical structures like the brainstem or spinal cord may be more difficult to remove completely^8^. In addition, different types of tumours may respond differently to various treatments, such as chemotherapy and radiation therapy^9^. Finally, factors such as the patientcs age, overall health, and the stage of the tumour can all impact treatment decisions and outcomes^6^.

## Diagnosing brain and spinal cord tumours

Diagnosing brain and spinal cord tumours requires a careful evaluation of the patient’s medical history and symptoms, as well as a variety of diagnostic tools and techniques. According to the American Brain Tumor Association, some common tests used to diagnose these tumours include imaging studies such as MRI and computed tomography scans, as well as biopsies and other laboratory tests^7^. The accuracy of the diagnosis can depend on several factors, including the size and location of the tumour, as well as the skill and experience of the healthcare provider. For example, small tumours or tumours located in hard-to-reach areas may be more difficult to detect with imaging studies alone^8^. In addition, some tumours may mimic other conditions or have similar symptoms, which can make accurate diagnosis more challenging^6^. Furthermore, some brain and spinal cord tumours are more rare or difficult to diagnose than others. For instance, some tumours may be undetectable during imaging studies, which can lead to delays in diagnosis and treatment^9^. In certain cases, more specialized imaging studies like PET scans or functional MRI may be employed to assess tumour activity or functional areas affected by the tumour^10^. Additionally, genetic testing can play a crucial role in diagnosing specific brain and spinal cord tumours, as it can identify genetic mutations or alterations that contribute to tumour development or progression. These genetic tests may include analysis of specific genes, such as IDH1 and IDH2 in gliomas, to provide valuable information for diagnosis and treatment planning^11^. The utilization of these advanced diagnostic tools and techniques enhances the accuracy of diagnosis, facilitates personalized treatment approaches, and contributes to improved patient outcomes.

## Treatment approaches

Treatment approaches for brain and spinal cord tumours include surgery, radiation therapy, and chemotherapy^12^. Each approach has its own benefits and drawbacks, and the choice of treatment will depend on the type and location of the tumour, as well as the patient’s individual needs and preferences^13^.

One notable advancement in the realm of surgical therapies for brain and spinal cord tumours is fluorescence-guided surgery (FGS). FGS employs fluorescent markers to enhance tumour visualization and assist in their complete removal. In a study by Palmieri *et al.* ^14^, they demonstrated that 5-ALA-guided FGS increased the rate of gross-total resection and improved progression-free survival in patients with high-grade gliomas.

Another innovative approach is robot-assisted neurosurgery, which is being increasingly utilized to offer enhanced precision and potentially better patient outcomes. A study highlighted the successful application of robotic stereotactic assistance in the biopsy and resection of deep-seated brain lesions^15^. This technique allows for more precise and controlled movements during surgery, minimizing the risk to surrounding healthy tissue.

Laser interstitial thermal therapy (LITT) is another evolving technique being used for treating brain and spinal cord tumours. LITT is a minimally invasive neurosurgical procedure that utilizes laser energy to heat and destroy tumour tissue. A research) showed promising results of LITT in the treatment of difficult-to-reach and recurrent brain tumours^16^.

Moreover, emerging treatment modalities like immunotherapy, targeted therapy, and gene therapy have shown promising results in the treatment of brain and spinal cord tumours. Immunotherapy harnesses the body’s immune system to target and destroy tumour cells, while targeted therapy aims to inhibit specific molecular targets involved in tumour growth. Gene therapy, on the other hand, involves introducing genetic material into cells to correct abnormalities or enhance the body’s natural defenses against tumours. These emerging treatment modalities hold great potential for improving patient outcomes and are actively being investigated and incorporated into clinical practice^17^.

These advancements in surgical techniques offer new possibilities for improved tumour visualization, precision, and patient outcomes in the treatment of brain and spinal cord tumours.

## Emerging therapies and future directions

In addition to the existing treatment approaches, emerging therapies and new treatments are being developed for brain and spinal cord tumours. Immunotherapies, such as immune checkpoint inhibitors and CAR-T-cell therapy, have gained attention for their potential in treating these tumours. Immune checkpoint inhibitors like pembrolizumab and nivolumab have shown efficacy in certain types of brain tumours by blocking inhibitory signals and enhancing the immune response against cancer cells^11,18^. However, challenges remain in identifying the most appropriate patient populations, optimizing treatment regimens, and managing potential immune-related adverse events associated with these therapies^19^. Similarly, while CAR-T-cell therapy has shown promise in clinical trials for glioblastoma, its successful implementation faces obstacles such as the need for improved targeting of tumour-specific antigens, managing potential toxicities, and addressing the immunosuppressive tumour microenvironment^20,21^.

Targeted therapies have also revolutionized the treatment landscape for certain types of brain tumours. For example, targeted therapies like ivosidenib and enasidenib have shown promise in inhibiting the mutant IDH1 protein, which drives tumour growth in glioblastoma cases with IDH1 gene mutations^11^. Despite their efficacy in specific subsets of patients, challenges remain in identifying predictive biomarkers, overcoming resistance mechanisms, and developing strategies to tackle intra-tumoral heterogeneity^22^. Additionally, the use of targeted therapies such as bevacizumab, although effective in some cases, requires further investigation to address concerns related to potential drug resistance and long-term outcomes^23,24^.

Gene therapies have recently emerged as a potential treatment strategy for spinal cord and brain tumours. In some clinical trials, gene therapies using oncolytic viruses have been investigated, but obstacles such as achieving optimal viral delivery, managing immune responses, and addressing potential off-target effects still need to be overcome^25^. Furthermore, while gene editing techniques like CRISPR-Cas9 offer exciting possibilities for directly modifying genetic abnormalities associated with these tumours, the development of safe and efficient delivery systems and ensuring precise targeting of gene editing tools are ongoing challenges^26^.

The ongoing development of these emerging therapies holds significant promise for the field of neuro-oncology. They offer the potential for more effective and personalized treatment options for patients with brain and spinal cord tumours. However, continued research, clinical trials, and advancements in these therapies are necessary to further validate their efficacy, optimize their safety, and overcome the challenges or limitations they may present. The collaboration between researchers, clinicians, and industry partners is crucial for advancing these therapies and improving patient outcomes in the future.

## Living with brain and spinal cord tumours

Living with brain and spinal cord tumours can present numerous challenges for patients and their families. These challenges may include physical, cognitive, and emotional symptoms, as well as financial burden and social isolation^27^. Supportive care is critical in managing the symptoms and side effects of treatment^28^. This includes medications, rehabilitation, and psychosocial support^27^. For example, organizations like the Brain Tumor Support and Education Group and the Spinal Cord Tumor Association provide valuable resources, educational materials, and support networks for patients and their families. Rehabilitation strategies, such as physical therapy, occupational therapy, and speech therapy, can also play a significant role in helping patients regain or maintain their functional abilities and improve their quality of life^29^. Additionally, patient education and self-advocacy play an important role in managing these tumours^30^. Patients and their families should be informed about treatment options, potential side effects, and strategies for coping with the impact of the disease on their lives^27^. By actively engaging in their care, patients can improve their quality of life and enhance their ability to manage their disease^30^.

## Conclusion

In conclusion, recent advancements in immunotherapies, targeted therapies, and gene therapies provide hope for improved treatment outcomes in patients with brain and spinal cord tumours. These emerging therapies offer more effective and personalized options, addressing treatment resistance and challenging tumor locations. Continued research and collaborative efforts are needed to further advance these therapies and improve patient outcomes. Comprehensive supportive care remains essential for managing the challenges associated with these tumours. Overall, these advancements offer promise for better treatment options and enhanced patient care.

## Ethical approval

Given the nature of the article, Narrative review, no ethical approval was required.

## Consent

No consent was required.

## Sources of funding

No funding was required.

## Author contribution

All authors contributed to this manuscript. A.M., A.T., M.M., A.S.: writing original draft, reviewing and editing. S.S.: supervision.

## Conflicts of interest disclosure

The authors declare that they have no conflicts of interest.

## Research registration unique identifying number (UIN)

Not needed.

## Guarantor

Dr. Abdallah Mansour.

## Data availability statement

The datasets generated and/or analyzed during the current study are publicly available.

## Provenance and peer review

Not commissioned, externally peer-reviewed.
